# Outlining tectonic inheritance and construction of the Min Shan region, eastern Tibet, using crustal geometry

**DOI:** 10.1038/s41598-017-14354-4

**Published:** 2017-10-23

**Authors:** Xiao Xu, Rui Gao, Xiaoyu Guo, Wenhui Li, Hongqiang Li, Haiyan Wang, Xingfu Huang, Zhanwu Lu

**Affiliations:** 1 School of Earth Sciences and Engineering, Sun Yat-sen University, Guangzhou, 510275 China; 2 Institute of Geology, Chinese Academy of Geological Sciences, Beijing, 100037 China

## Abstract

The ongoing collision between India and Eurasia has created the Tibetan Plateau, which features high elevations and large crustal thicknesses. The easternmost portion of the plateau has long been a key region for studying the uplift mechanism of the Tibetan Plateau, especially after the 2008 *Ms*. 7.9 Wenchuan earthquake. However, previous studies have assumed that easternmost Tibet is tectonically homogeneous, and the tectonic significance of the Min Shan has been overshadowed by that of its more conspicuous neighbour, the Longmen Shan region. Here, we describe the crustal geometry of the Min Shan region using two newly obtained deep seismic reflection profiles. In this study, we identify an upper-lower crust mechanical decoupling within the Min Shan region; the Min Shan region is tectonically delineated by an inherited boundary fault zone, the Huya fault zone, which was responsible for triggering the 2017 Jiuzhaigou *M* 7.0 earthquake. Together with the gravity dataset and previous studies in this area, the outlined crustal geometry indicated that crustal-scale shortening at the eastern plateau margin is a primary mechanism driving uplift, although extensive uplift might have occurred due to the decoupled shortening between the upper and lower crust.

## Introduction

Uplift of the Tibetan Plateau is one of the most fascinating geological puzzles in the world. It represents the widespread intracontinental response to the ongoing India-Eurasia collision that began ~50 Myr ago^1,2^, The tectonic responses along the plateau margins to the outward growth of the plateau remain debated^3–7^. Two end member models, i.e., continuous crustal deformation and lower crustal flow^8,9^, have been developed based on geological and geophysical studies in Tibet^10–13^. The critical difference between these two models is the vertical variation in response to contraction, particularly in the lower crust. The Moho offset and lower crustal flexure are not expected based on crustal flow model studies, and continuous crustal deformation has resulted in coupled mechanics between the upper and lower crust.

**Figure 1:**
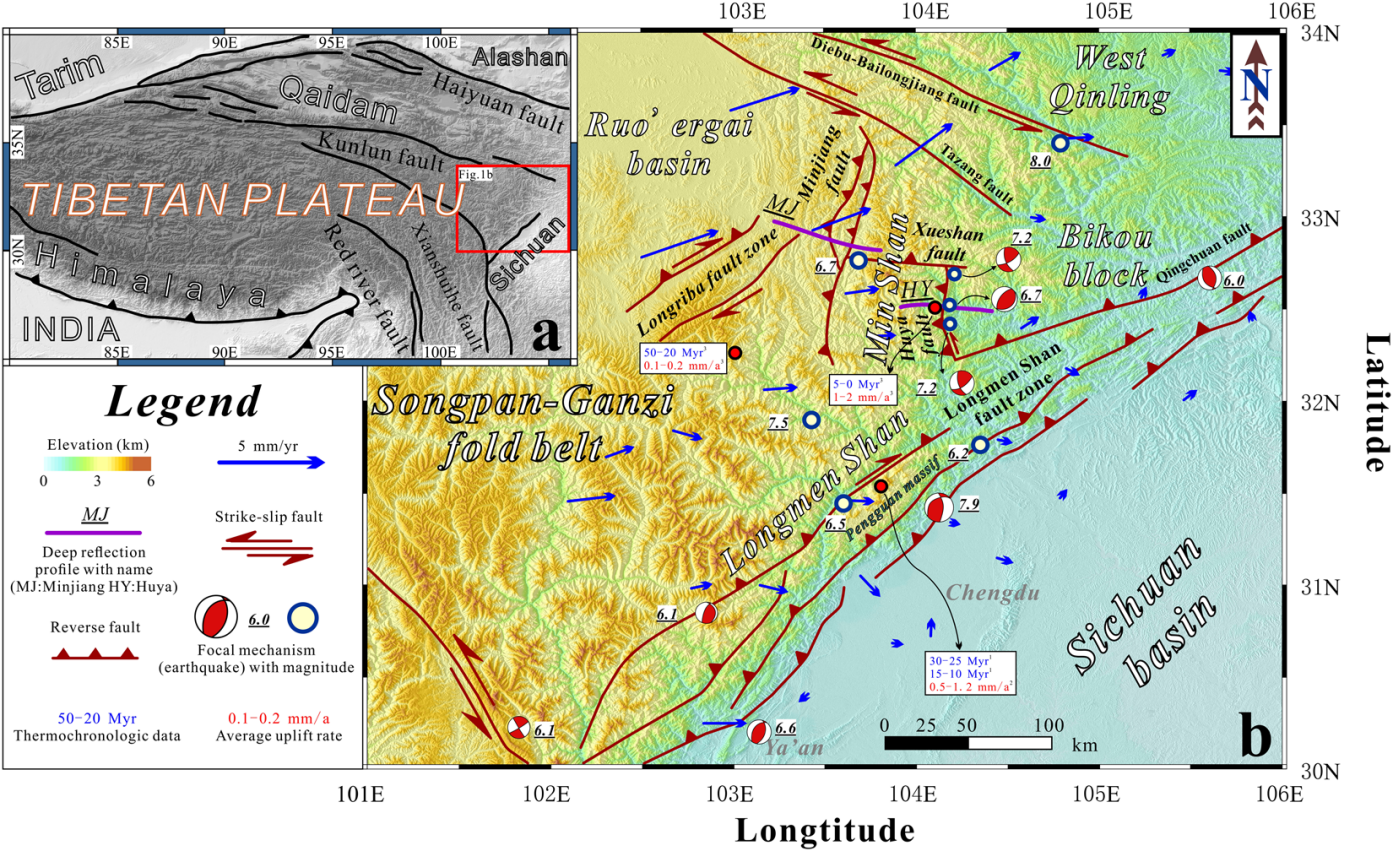
Simplified regional tectonic map of the eastern margin of the Tibetan Plateau. (**a**) Map showing the topography of the Tibetan Plateau and the location of Fig. 1b. (**b**) A simplified structural map and shaded topographic relief^3,10,11^. GPS measurements^22^ indicating current crustal deformation; the Min Shan and Longmen Shan regions are depicted by a primarily northeastward extrusion. Based on the thermochronologic data^11,19,20^, the Min Shan region has the highest uplift rate. The digital elevation data were downloaded from http://srtm.csi.cgiar.org/SELECTION/inputCoord.asp. The topographic map was prepared using Generic Mapping Tools (GMT)^36^ (http://gmt.soest.hawaii.edu/). The map was drafted by X.X. using the CorelDRAW software package (http://www.coreldraw.com/en/product/graphic-design-software/).

The lateral extrusion of the Tibetan Plateau is widely accepted to be resisted by the rigid Sichuan basin to the east, resulting in the uplift of the Longmen Shan region and the Min Shan region^14^ (Fig. 1). The disastrous Wenchuan and Lushan earthquakes^15^ provided a reminder that the Longmen Shan, which has extremely high relief and a low shortening rate, accommodates the uplift and lateral extrusion of the Tibetan Plateau^3,16^. However, previous studies have considered the eastern Tibetan Plateau to be a tectonically uniform unit and have ignored the Min Shan region in the northernmost portion of the eastern margin of the Tibetan Plateau, which possesses the same topographic relief as the Longmen Shan and experiences frequent intrablock earthquakes^17^, e.g., the most recent 2017 *Ms*. 7.0 Jiuzhaigou earthquake.

The Min Shan region is located northeast of the Longmen Shan (Fig. 1). It represents a geological and structural margin of eastern Tibet^10^ and has an average elevation of over ~3500 m. The present-day horizontal shortening rate is less than 2–3 mm/yr, and several disastrous earthquakes have occurred on the Minjiang and Huya faults^18^. The Min Shan region differs from the Longmen Shan region in terms of the uplift rate and age. The thermal histories provided by previous studies indicate slow cooling in eastern Tibet during the early Jurassic to mid-Tertiary^19^. Based on the inferred cooling in the Min Shan region, the average uplift rate during the last 5 My has been 1–2 mm/yr^19^. In addition, the Longmen Shan experienced uplift earlier, with two phases of growth occurring before 10 Ma^20^ and an estimated uplift rate of ~0.5–1.2 mm/yr^11,19,20^. These data indicate that the eastern Tibetan Plateau is tectonically heterogeneous and represents an important natural laboratory for studying the crustal response to mountain building at the margins of the plateau.

The Min Shan region is bounded by the Minjiang fault zone to the west and the Huya fault zone to the southeast (Fig. 1). Geological field data and earthquake focal mechanisms show that both fault zones consist of west-dipping reverse faults^18^. The Minjiang fault zone is connected with the Huya fault by the Xue Shan fault. It further interacts with the Tazang fault to the north^21^ and with the Longmen Shan fault zone to the south^11^. These faults generally follow the boundary of the Paleozoic sediments (the Bikou block) and Triassic flysch (the Ruo’ergai basin). However, under the same stress field and strain, e.g., folding, in the Ruo’ergai basin, the Min Shan region and the Bikou block have different characteristics. In addition, the existence of the Minjiang and Huya faults indicates a different crustal strength between the Bikou block and the adjacent Min Shan region.

The western flank of the Min Shan contains Quaternary strata that dip ~10° to the northwest^18^, indicating that the Min Shan accommodated east-verging contraction, but the current shortening rate could not have produced this feature^18^. According to GPS observations^22^, the focal mechanisms of earthquakes occurring along the Huya fault and the geometry of the faults^18^, right-lateral movement along the Wenchuan-Maowen fault is transferred to the Qingchuan fault through the Min Shan. The Min Shan region, which acts as the transition zone between the Kunlun fault and the Longmen Shan fault zone^17^, is a key area in understanding northeastward extrusion at the eastern edge of the Tibetan Plateau. To gain additional insights into the deep structural framework of the Min Shan region, two deep seismic reflection profiles were collected in this region in 2014 (Fig. 1). Regional gravity anomaly data are employed to help understand the regional deformation. This large dataset is crucial for imaging the crust in unprecedented detail and helps characterize both the uplift and the current eastern boundary of the plateau.

## Results

### Decoupled upper and lower crust of the Min Shan region

**Figure 2:**
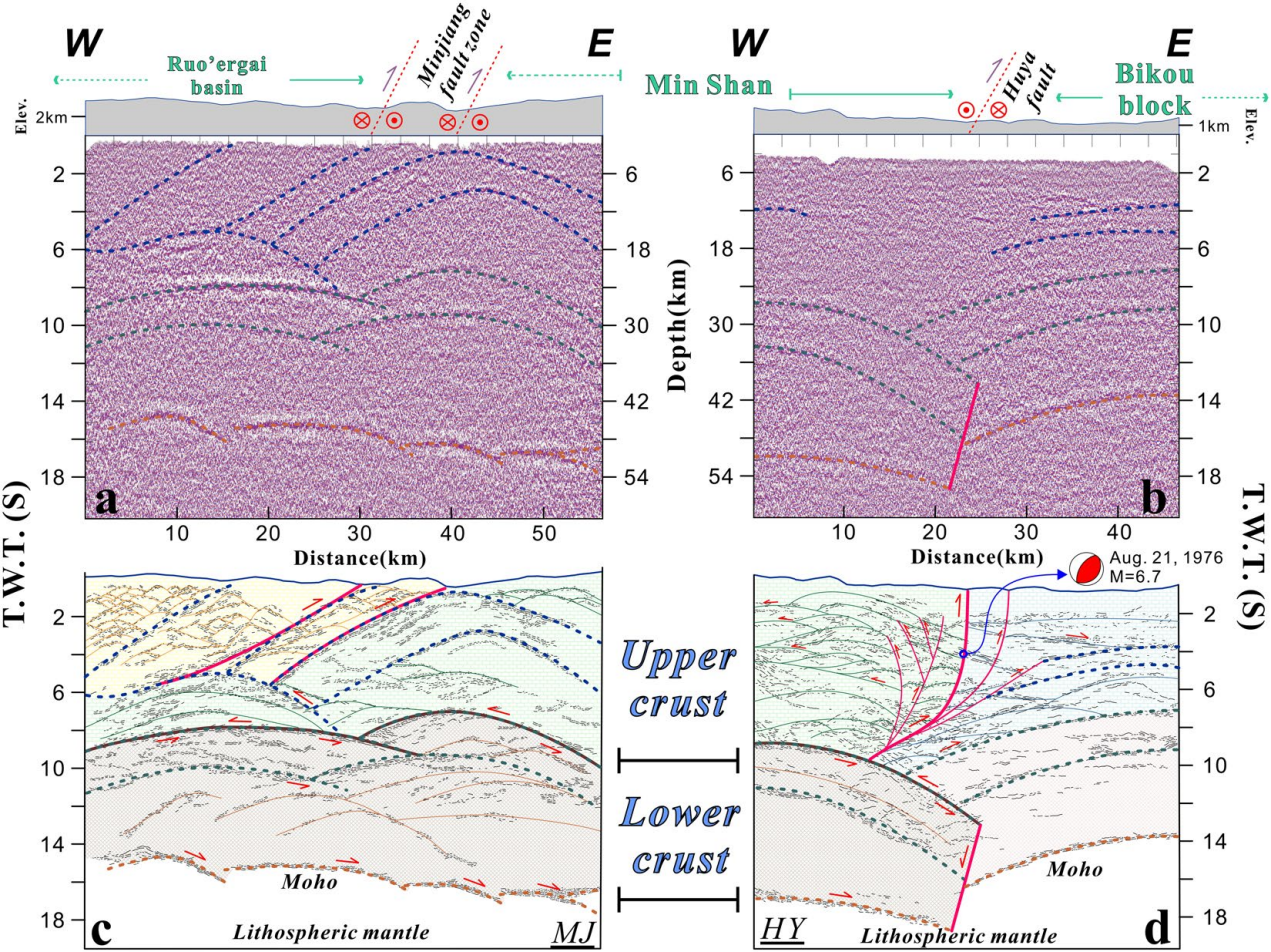
Deep seismic profiles. Please see Fig. 1b for the locations of these profiles. (**a**) Original profile crossing the Minjiang fault zone with generalized interpretations of the strata. (**b**) Original profile crossing the Huya fault with generalized interpretations of the strata. (**c**) Interpreted profile crossing the Minjiang fault zone. The red lines show the location of the Minjiang fault zone at depth, and the arrows indicate the relative directions of motion. (**d**) Interpreted profile crossing the Huya fault zone. The Huya fault has been divided into two segments by the decoupled crust underlying the Min Shan. The deformation is consistent between the upper and lower crust within the Bikou block. The figure was drafted by X.X. using the CorelDRAW software package (http://www.coreldraw.com/en/product/graphic-design-software/).

We present a new characterization of the crustal structure in eastern Tibet based on new controlled-source seismic data collected in 2014 and provide new insights into the mechanism of deep crustal deformation responsible for the observed uplift. According to tectonic settings along the profiles and offsets of reflectors, the seismic reflection profiles (Fig. 2a,b) show three domains identified in the seismic reflection data. The identified subdomains are the Ruo’ergai basin (northwest of the Minjiang fault zone), the Min Shan block (the zone between the Minjiang fault zone and the Huya fault), and the Bikou block. The topographic surface in the Ruo’ergai basin has unusually low relief, but high relief appears to the southeast of the Minjiang fault zone. Therefore, we define the Min Shan block as a convergent zone between the eastern Songpan-Ganzi terrane (Ruo’ergai basin) and the Bikou block.

In detail, both the upper crust and lower crust beneath the Ruo’ergai basin (Songpan-Ganzi terrane) have been folded, but the folds have different vertical scales (Fig. 2c). To the west of the Minjiang fault zone, a bright spot that extends downward to depths of 6–8 s (t.w.t.) was detected at the bottom of the upper crust beneath the Ruo’ergai basin (Fig. 2a). Similar features observed in southern Tibet^23^ have been interpreted as the result of partial melting at mid-crustal depths^24^. Our seismic reflection data clearly document the different types and degrees of deformation in the upper and lower crust beneath the Ruo’ergai basin and the Min Shan region (Fig. 2a,c). Thus, we interpret a decoupled crust under compression from the west and further propose that the decoupled motion might have caused partial melting on top of the lower crust. The Minjiang fault zone is both a topographic and upper crustal boundary, and it developed during the lateral growth of the plateau. The fold system dominates the entire crust of the Min Shan region, and the degree of folding decreases with depth towards the lower crust (Fig. 2c). The reflection features in the Huya profile (Fig. 2b,d) are relatively simple, but they contain important information. The Huya fault defines the intrablock boundary between the Bikou block and the Min Shan. This fault has been divided into two segments that reflect the different responses of the upper and lower crust. The behaviour of the Bikou block is similar to that of the Sichuan basin, which is rigid and relatively stable. Thus, in response to crustal shortening and the resistance of the rigid Bikou block, a ductile detachment fault developed, along which the upper crust was thrusted upward and the lower crust was bent downward. Crustal folding triggered the uplift of the Min Shan, but this folding had different scales on the upper and lower crust. The seismic reflectors in the Min Shan profile indicate that crustal shortening occurred in both the upper and lower crust but that the upper and lower crust thrusted in opposite directions.

### Regional crustal deformation of the eastern Tibetan Plateau

**Figure 3:**
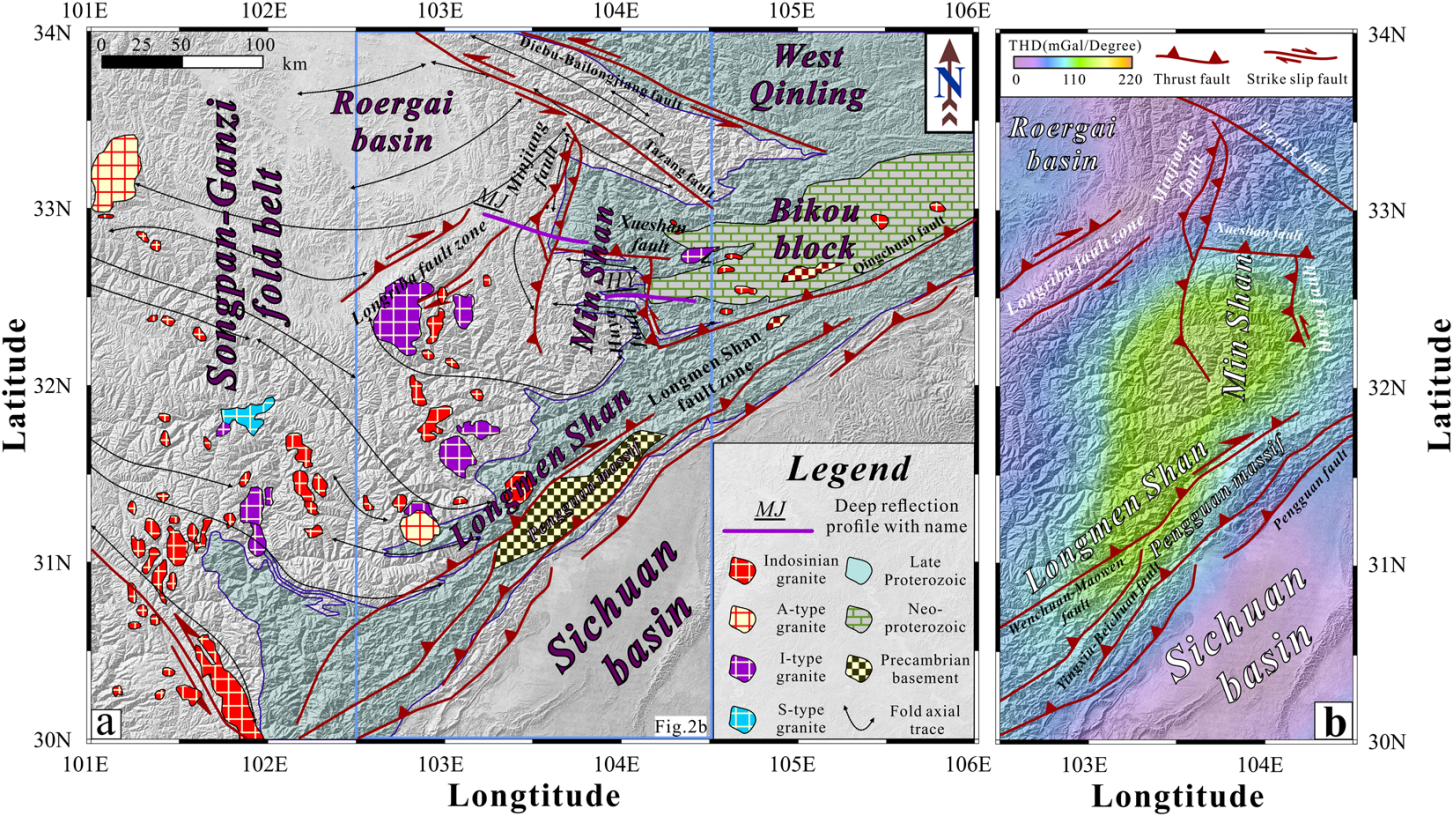
Regional geological map and the total horizontal derivative (THD) of the simple Bouguer anomaly. (**a**) Topographic map indicating the extents of tectonostratigraphic terranes and granite bodies^28–30^. The generalized geological map of the research area was modified after The Geology Map of China [1:2,500,000]. Primarily Neoproterozoic metamorphic rocks are exposed in the Bikou block, and the granites exposed in the southwestern Songpan-Ganzi terrane include A, I and S types. (**b**) Except for the rigid Sichuan basin, the Minjiang fault zone and Longriba fault zone are associated with smaller THD values of the simple Bouguer anomaly than other areas shown on this map. These observations may indicate that the strain within the crust is released through the faults. Therefore, the Huya fault and Longmen Shan fault zones still pose a considerable risk of disastrous earthquakes. The digital elevation data were downloaded from http://srtm.csi.cgiar.org/SELECTION/inputCoord.asp. The topographic map was prepared using Generic Mapping Tools (GMT) (http://gmt.soest.hawaii.edu/). The map was drafted by X.X. using the CorelDRAW software package (http://www.coreldraw.com/en/product/graphic-design-software/).

To understand the regional crustal deformation over larger scales, gravity anomaly data were applied. The total horizontal derivative (THD) of the gravity anomaly highlights the shape of the western margin of the Yangtze block between the Longriba fault zone and the Longmen Shan fault zone (Fig. 3b). According to the GPS observations^22^ and the distribution of earthquakes^18^, this passive margin indicates that the magnitude of deformation differs according to its surroundings. Previous studies have shown that the deep structure of the continental granitic to dioritic crust in orogenic belts includes a strong, brittle upper layer and a ductile, deformable lower layer^25^. The Songpan-Ganzi terrane has experienced multiple tectonic events since the late Palaeozoic^26^. Before the India-Asia collision, the major tectonic event experienced by the Songpan-Ganzi terrane was the closure of the Palaeo-Tethys ocean^27,28^. This terrane is bordered by the Qaidam basin and the North China block to the north, the South China block to the east, and the Qiangtang terrane and the Yidun arc terrane to the south. Syntectonic granitoids indicate that the Songpan-Ganzi terrane experienced a post-orogenic stage^29,30^ (Fig. 3a). Based on the distribution and age of the granites, the eastern Songpan-Ganzi terrane and the western Yangtze block experienced a second phase of crustal thickening during the Cenozoic. In the Min Shan region, Mesozoic granites are absent, indicating less intense deformation in this region compared to that of the Longmen Shan region. Therefore, the crustal deformation revealed by the THD of the gravity anomaly was produced by the latest Cenozoic tectonic event. By this time, the crustal deformation in the Min Shan region had become more intense than that of the Longmen Shan.

## Discussion

### The 2017 *M* 7.0 Jiuzhaigou earthquake

**Figure 4:**
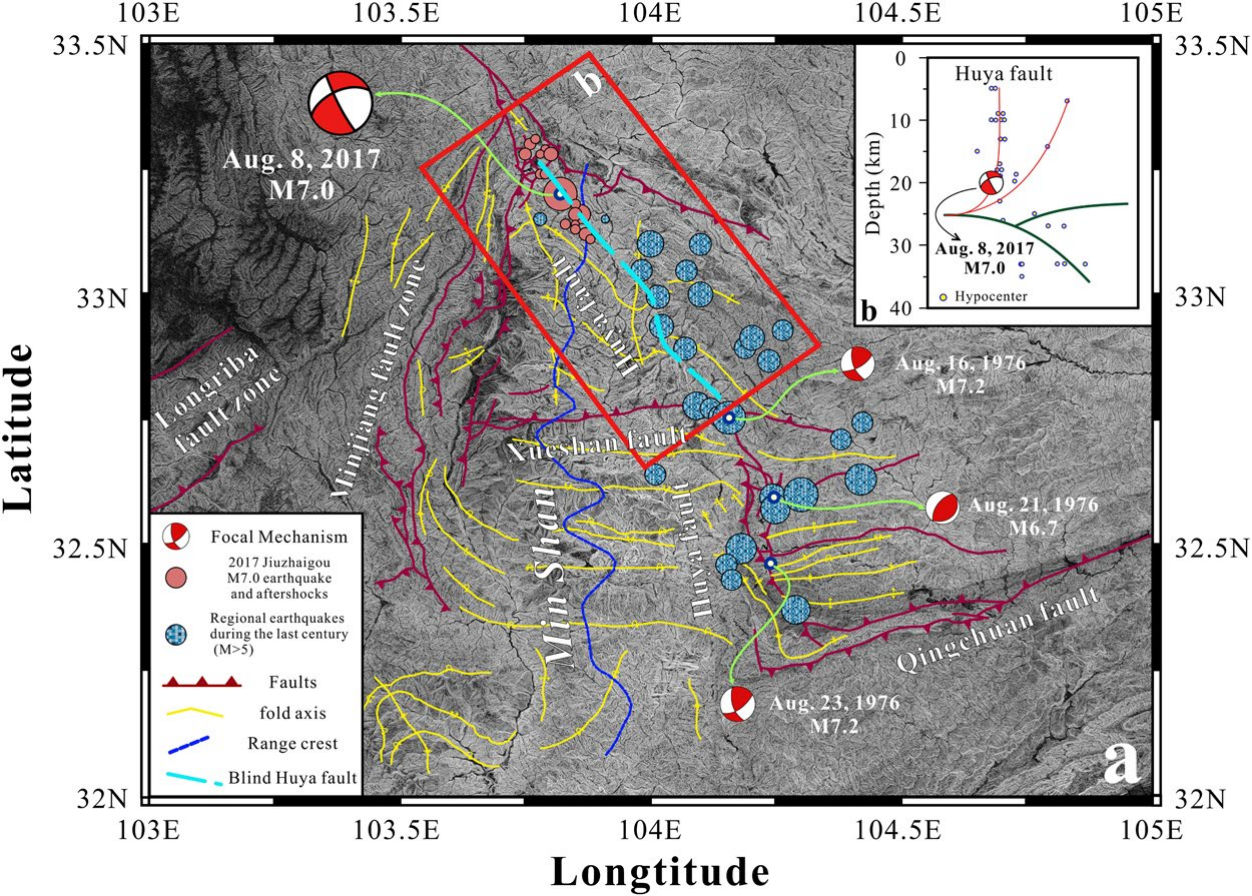
Detailed structural map of the Min Shan region. (**a**) A detailed structural map^18^ over shaded topographic relief. 2017 Jiuzhaigou earthquakes (Hypocenters from the China Earthquake Networks Center; focal mechanism from the USGS) and regional earthquakes during the last century (magnitude ≥ 5; http://service.iris.edu/fdsnws/event/docs/1/builder/) along the Huya fault are shown. The red box shows the location of Fig. 4b. The digital elevation data were downloaded from http://srtm.csi.cgiar.org/SELECTION/inputCoord.asp. (**b**) Projected Hypocenters in the red box (Fig. 4a) along the main strike of the blue dashed line in Fig. 4a. The red lines are interpreted as the Huya fault; the green lines are strata. The topographic map was prepared using Generic Mapping Tools (GMT) (http://gmt.soest.hawaii.edu/). The map was drafted by X.X. using the CorelDRAW software package (http://www.coreldraw.com/en/product/graphic-design-software/).

On August 8^th^, 2017, an *M* 7.0 earthquake occurred in the northern Min Shan region (Fig. 4a). The event occurred in a complex region that possesses three potential faults: the Minjiang fault zone, the Tazang fault and the Huya fault (Fig. 4a). The focal mechanism solution indicates that the earthquake could have been triggered by either a northwest-striking left-lateral fault or on a northeast-striking right-lateral fault (U.S. Geological Survey, Fig. 4a). Thus, to better understand what triggered the earthquake, we plot the main shock and aftershocks (red dots in Fig. 4a), which are distributed NW-SE and are coincident with the strike of a fault zone (blue dashed line in Fig. 4a) that can be recognized from the highly resolved topographic relief map. In addition, surface geological investigations^18^ have identified a sequence of surface curvatures that bend consistently towards the south (yellow line in Fig. 4a). They are terminated by the Xueshan Fault in the south, which is depicted as a south-verging thrust fault. However, the plotted historic earthquakes (blue dots in Fig. 4a) are not terminated but can be continuously traced beyond the Xueshan fault zone to the south, where they coincide with the Huya fault zone. Additionally, similar surface curvature is evident on both sides of the Huya fault zone. Thus, together with the hypocentres of approximately 1976 aftershocks in the northern Xushan fault zone (Fig. 4b), the similar surface structures allow us to determine that the fault in the northern Xueshan fault zone is the northward extent of the Huya fault zone, which triggered the *Ms* 7.0 Jiuzhaigou earthquake.

### Crustal geometry

**Figure 5:**
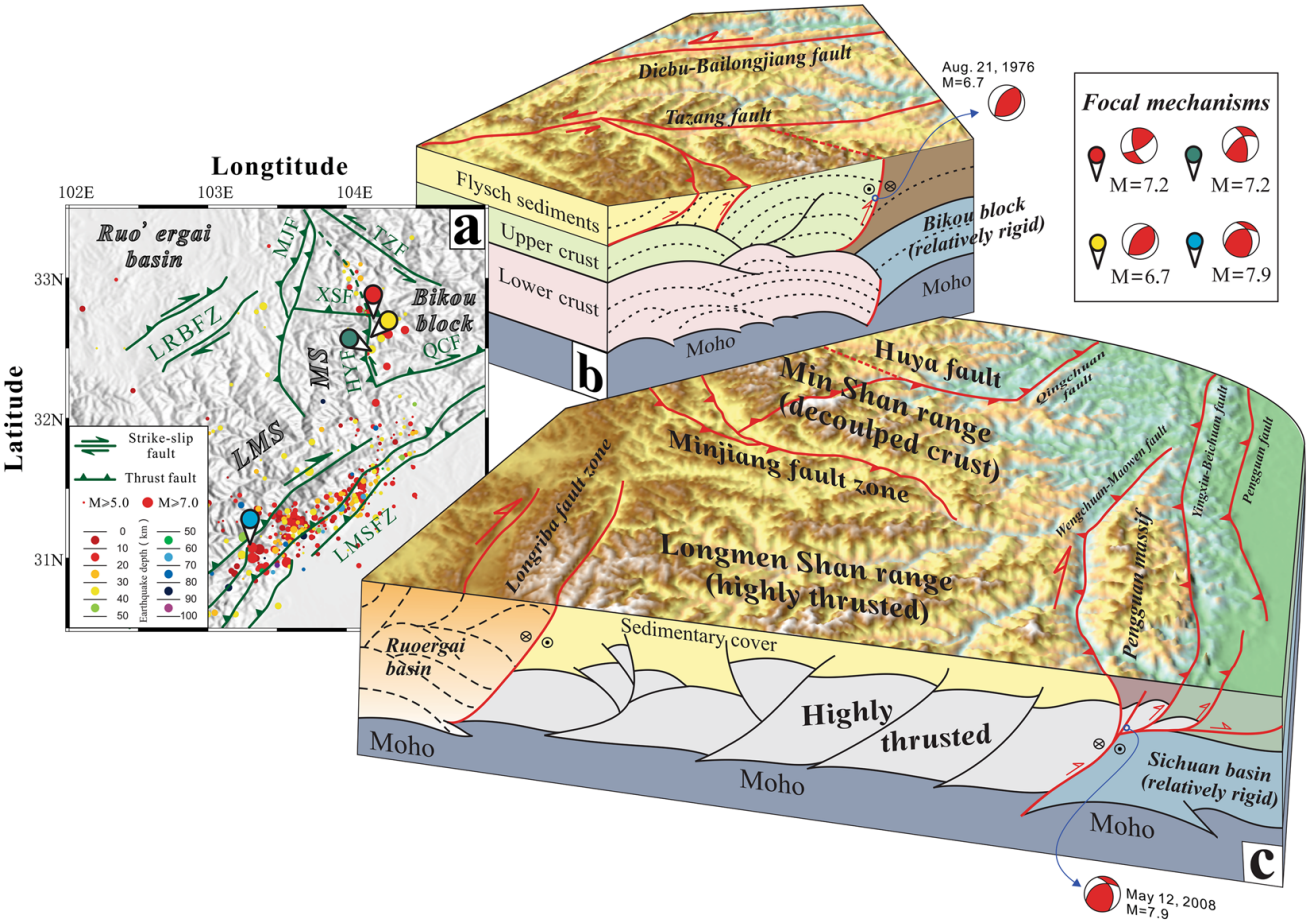
Earthquake distribution and sketches of the deep structure of the Min Shan and Longmen Shan regions. (**a**) Regional earthquakes during the last century (magnitude ≥ 5; http://service.iris.edu/fdsnws/event/docs/1/builder/). The Wenchuan earthquake^3^ and the Songpan-Pingwu earthquake^18^ are labelled. (**b**) The crustal structure of the Min Shan and the Bikou block. (**c**) The crustal structure of the Longmen Shan and the Sichuan basin^10^. The digital elevation data were downloaded from http://srtm.csi.cgiar.org/SELECTION/inputCoord.asp. The topographic map was prepared using Generic Mapping Tools (GMT) (http://gmt.soest.hawaii.edu/). The map was drafted by X.X. using the CorelDRAW software package (http://www.coreldraw.com/en/product/graphic-design-software/).

In summary, our interpretation of the deep crustal structures (Fig. 5) in the Min Shan indicates that crustal folding due to decoupled deformation between the upper and the lower crust is a primary mechanism for generating uplift in the Min Shan region. Moreover, systems of folds and faults are fully developed in the upper and lower crust. However, at the range front along the Huya fault, the lower crust has bent against the Bikou block, whereas decollement folding and thrusting have developed in the upper crust. Thus, our interpretation reveals the strata and deformation in the deep crust within this area for the first time. No strong evidence from the eastern margin of the Tibetan Plateau supports the lower crustal flow model, which proposes that the lower crust is easily deformed and that the Moho should contain a downward bulge. These predictions are inconsistent with the deep structure observed along the Huya fault, where the Moho offset and lower crustal flexure appear (Fig. 2). The Huya fault zone can be viewed as a newly identified fault zone that marks the northeastern edge of the Tibetan Plateau.

### Uplift mechanism

To understand the uplift mechanism of the eastern Tibetan Plateau, we compared the deep crustal structures between the Min Shan and the Longmen Shan (Fig. 5). The crustal-scale thrust faults along the Longmen Shan range front accommodate crustal shortening, which is a fundamental mechanism driving uplift and crustal thickening. However, more accumulated uplift can be accommodated within decoupled crust. Another important unit that needs to be considered is the Pengguan massif, which is located between the central portion of the Longmen Shan fault zone and the southern end of the Min Shan fault. It is mainly delineated by the Yingxiu-Beichuan and Pengxian-Guanxian faults to the southeast and the Wenchuan-Maowen fault to the northwest (Fig. 3b). This body of late Precambrian autochthonous basement rock has been mainly exposed via thrusting and is overlain by Palaeozoic sedimentary rocks to the northeast and southwest along the Longmen Shan fault zone^5^. Based on the focal mechanism of the 2008 Wenchuan earthquake, the eastern bounding faults are well documented as northwest-dipping thrust faults with right-lateral slip. Combining our results with the earthquake distributions (Fig. 5a) indicates that this earthquake zone has a high potential for triggering large and very devastating events because of the overlapping compressional features.

## Methods

### Deep seismic reflection data

Two short deep seismic profiles were created across the Minjiang fault (55 km) and the Huya fault (45 km). The geophone group spacing was 50 m, and the recording time was 30 s for the small (36 kg) and medium (120 kg) sources and 60 s for the large sources (up to 500 kg). The recording geometry for the 36 kg and 120 kg shot sources provided 75-fold common midpoint (CMP) coverage for the processing. The seismic data processing was performed with a combination of the CGG, ProMAX, GeoTomo, GeoRest and GeoDenoise systems. The data processing was performed by conducting tomographic static correction, true-amplitude recovery, frequency analysis, filter-parameter tests, surface-consistent-amplitude corrections, surface-consistent de-convolution, coherent noise suppression, random noise attenuation, human-computer interactive velocity analysis, residual statics correction, Kirchhoff pre-stack time migration from rugged topography and post-stack polynomial fitting for removing noise. An iterative procedure was adopted to obtain the optimal parameters for stacking and post-stack noise attenuation. The relative true amplitudes were preserved by these processing steps. These high-density and near-vertical reflection data are the basis of our integrated study of the seismic structure of the crust in the study area.

### Gravity anomaly data

The gravity anomaly data were obtained from the International Centre for Global Earth Models (ICGEM; http://icgem.gfz-potsdam.de/ICGEM/ICGEM.html). The data were processed with the Geosoft/Oasis Montaj processing and analysis package. The regional simple Bouguer anomaly is strongly controlled by the crustal thickness in eastern Tibet^31^. To highlight the characteristics of structures within the crust, we removed the portion of the anomaly caused by variations in the topography of the Moho. The Moho depth was determined using the results of receiver function studies^32,33^. The regional residual of the crustal gravity anomaly is better correlated with the surface topography and better coincides with the thrust faults^34^. These thrust faults mainly correspond to the topographic boundary of the eastern plateau. The western edge of the Yangtze block is complex. The THD of the gravity anomaly can be used to detect the edges of different structures^35^.

## Electronic supplementary material

Supplementary information
