# Supplementary material for: Outlining tectonic inheritance and construction of the Min Shan region, eastern Tibet, using crustal geometry
Source: Sci Rep. 2017 Oct 23;7:13798. doi: 10.1038/s41598-017-14354-4 (PMC5653797; doi:10.1038/s41598-017-14354-4)
Supplement: Supplementary file 1 — Supplementary information [file 41598_2017_14354_MOESM1_ESM.docx]

**Outlining tectonic inheritance and construction of the Min Shan region, eastern Tibet, using crustal geometry**

**Xiao Xu^1^, Rui Gao^1,2*^, Xiaoyu Guo^2^, Wenhui Li^2^, Hongqiang Li^2^, Haiyan Wang^2^, Xingfu Huang^2^, Zhanwu Lu^2^**

**Affiliations:**

1. **School of Earth Science and Geological Engineering, Sun Yat-sen University, Guangzhou 510275, China**
2. **Institute of Geology, Chinese Academy of Geological Sciences, Beijing 100037, China**

***Correspondence to gaorui66@mail.sysu.edu.cn**

1. **Supplementary Figures**

Supplementary Figure S1. Unmigrated and migrated images of the Huya and Minjiang reflection profiles without superimposed interpretation.

Supplementary Figure S2. Details of the migrated images of the Huya seismic profiles with and without a superimposed interpretation.

Supplementary Figure S3. Crustal deformation in the Min Shan region.

**Supplementary Figure S1. | Unmigrated and migrated images of the Huya and Minjiang reflection profiles without superimposed interpretation.**

1. Unmigrated Huya stack profile.
2. Unmigrated Minjiang stack profile.
3. Post-stack time migration of the Huya reflection profile.
4. Post-stack time migration of the Minjiang reflection profile.

**
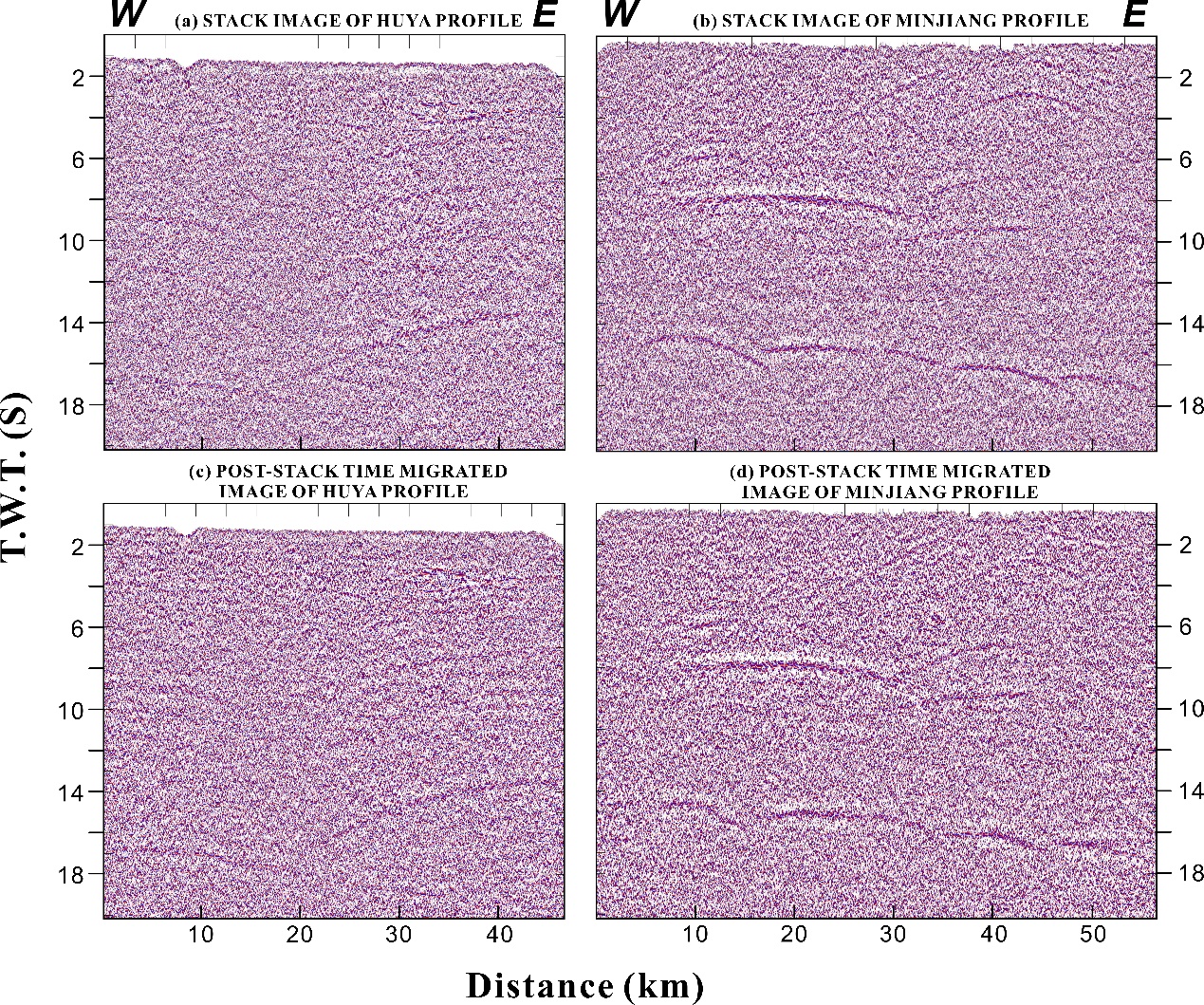
**

**Supplementary Figure S2. | Details of the migrated images of the Huya seismic profiles with and without a superimposed interpretation.**

1. The stratigraphy frame. The green triangles indicate the clear interfaces. Boxes with c and e indicate the locations of Figs. S2c and S2e.
2. The upper and lower crust with highlighted stratigraphy. Two blocks meet in the middle beneath the Huya fault on the surface. The Min Shan region is to the west, while the Bikou block is to the east. The east flank of the Min Shan region dips to the east, whereas the Bikou block dips to the west. The Min Shan region is vertically oriented, the upper crust is brittle, and it is fragmentized under compression. Thus, the reflection is fuzzy. The upper and lower crust of the Min Shan region is separated at about 8-9 s in two-way travel time (T.W.T.). Based on the depth and thickness of the lower crust in the Min Shan region, the lower crust of the Bikou block starts at 7 s in T.W.T., and the upper crust lies above it. The red dashed line indicates the Moho region, the green dash lines indicate the stratigraphy of the lower crust, and the blue lines show the stratigraphy of the upper crust. The box containing g indicates the location of Fig. S2g.
3. Details of the lower crust along the boundary between the Min Shan and Bikou blocks. The green triangles denote the same stratigraphy as in Fig. S2a; more details can be identified in this zoomed figure. Parallel layers are labelled with blue triangles.
4. Interpretation of the lower crust. The tips of the layers with two dipping directions in the lower crust are the contact points between the two blocks. The red line denotes the connection points. Based on the geometry and the location of the surface, it is interpreted as the lower part of the Huya fault.
5. Details of the upper crust along the boundary between the Min Shan and Bikou blocks. The green triangles denote the same stratigraphy as that in Fig. S2a. The blue angles denote visible reflections. The red lines are the Huya fault. The Huya fault is identified on the surface based on geological information; the fault is traced from the surface to deeper layers by connecting the reflection offsets. It ends at the lower crust of the Min Shan region.
6. Upper panel of the Huya fault. The small visible reflections show the fully grown Huya fault in the upper crust. The blue beach ball is the focal mechanism solution of the 1976 earthquake; the focus of this earthquake is on this profile.
7. Upper crust of the Min Shan region. Please see Fig. S2b for the location. The right panel is Fig. S2f. The blue angles denote visible reflections.
8. Interpretation of the upper crust in the Min Shan region. The details of the upper crust in the Min Shan region show that the main deformation is a thrust fault fold.


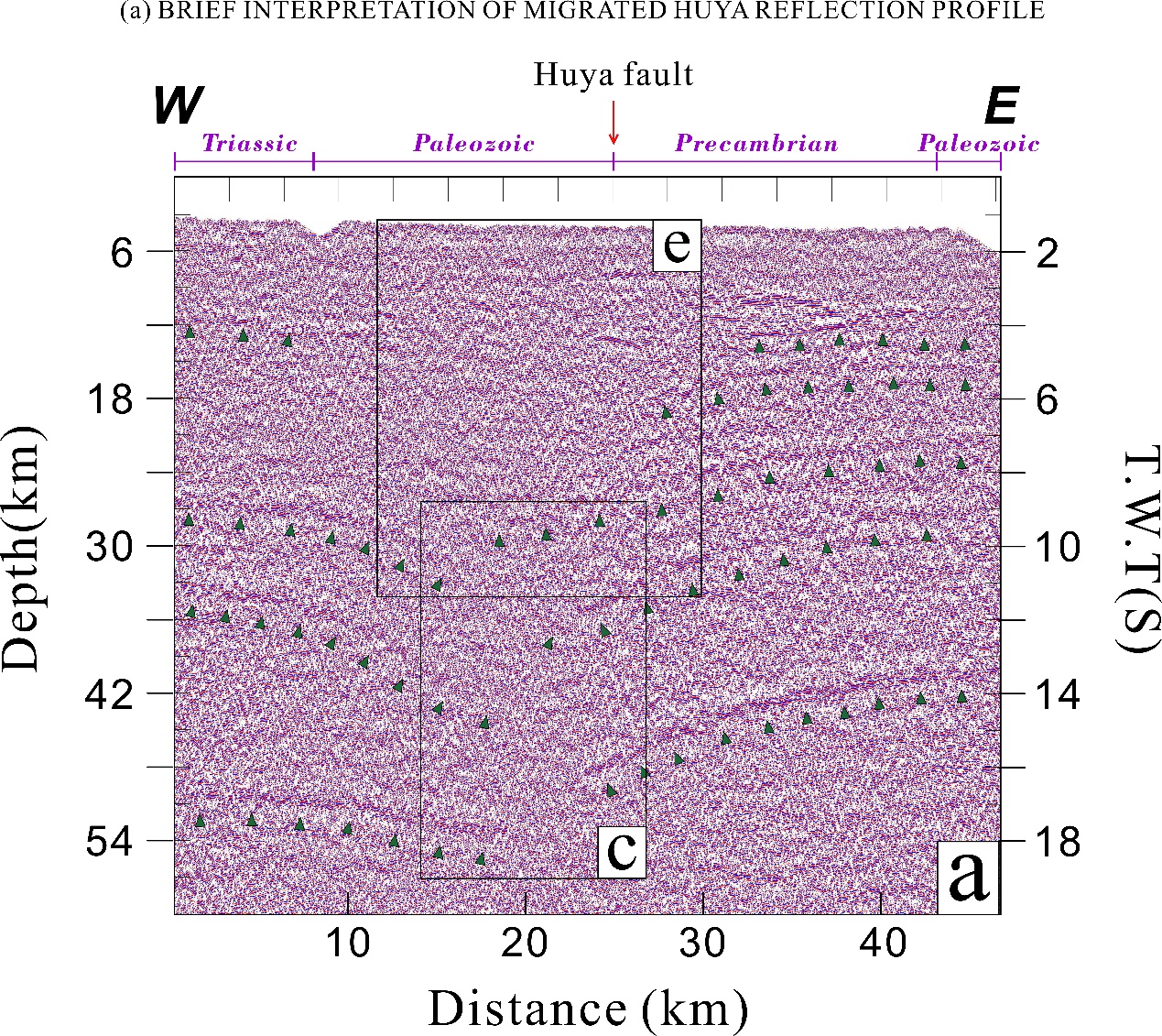


**
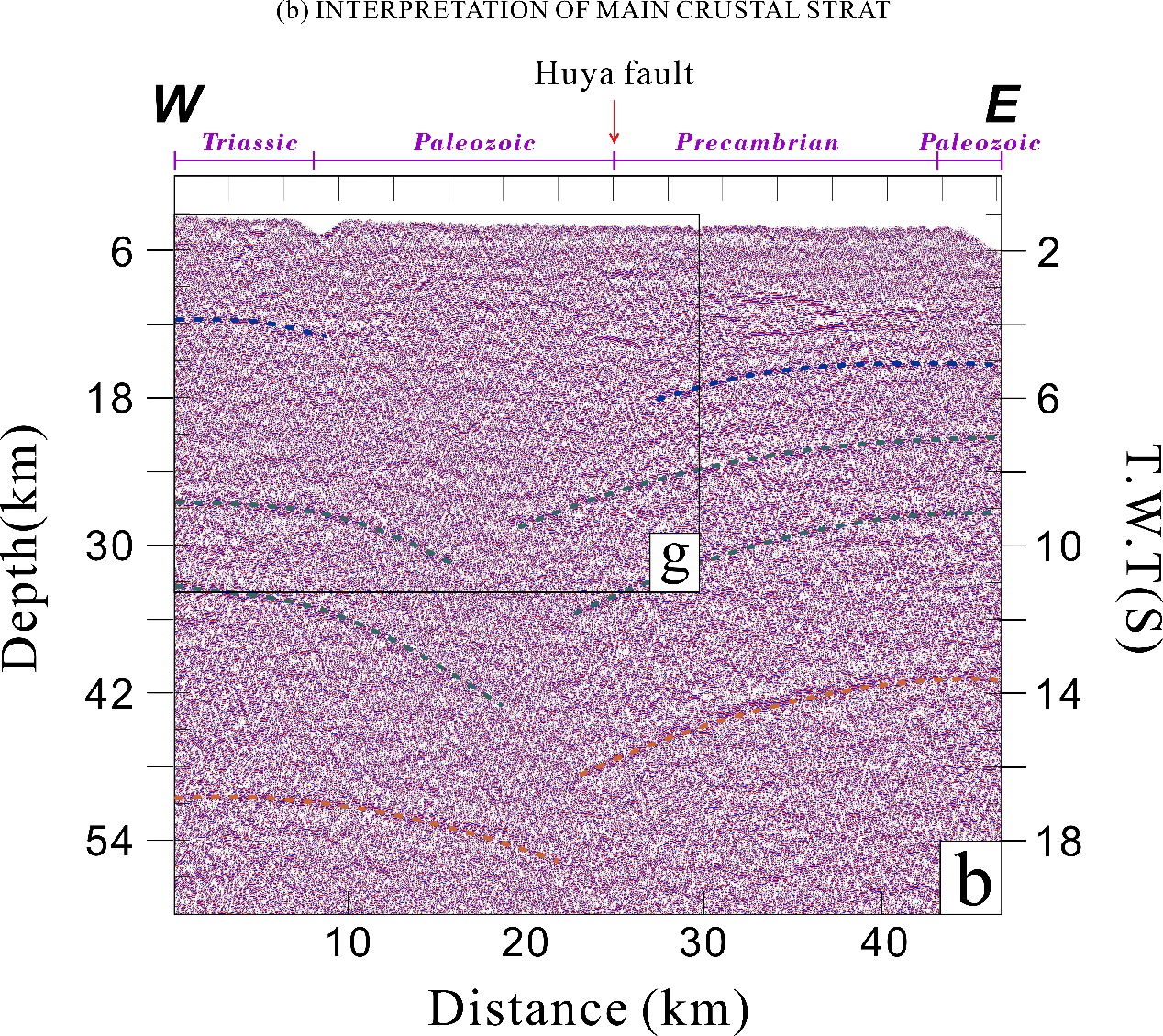
**

**
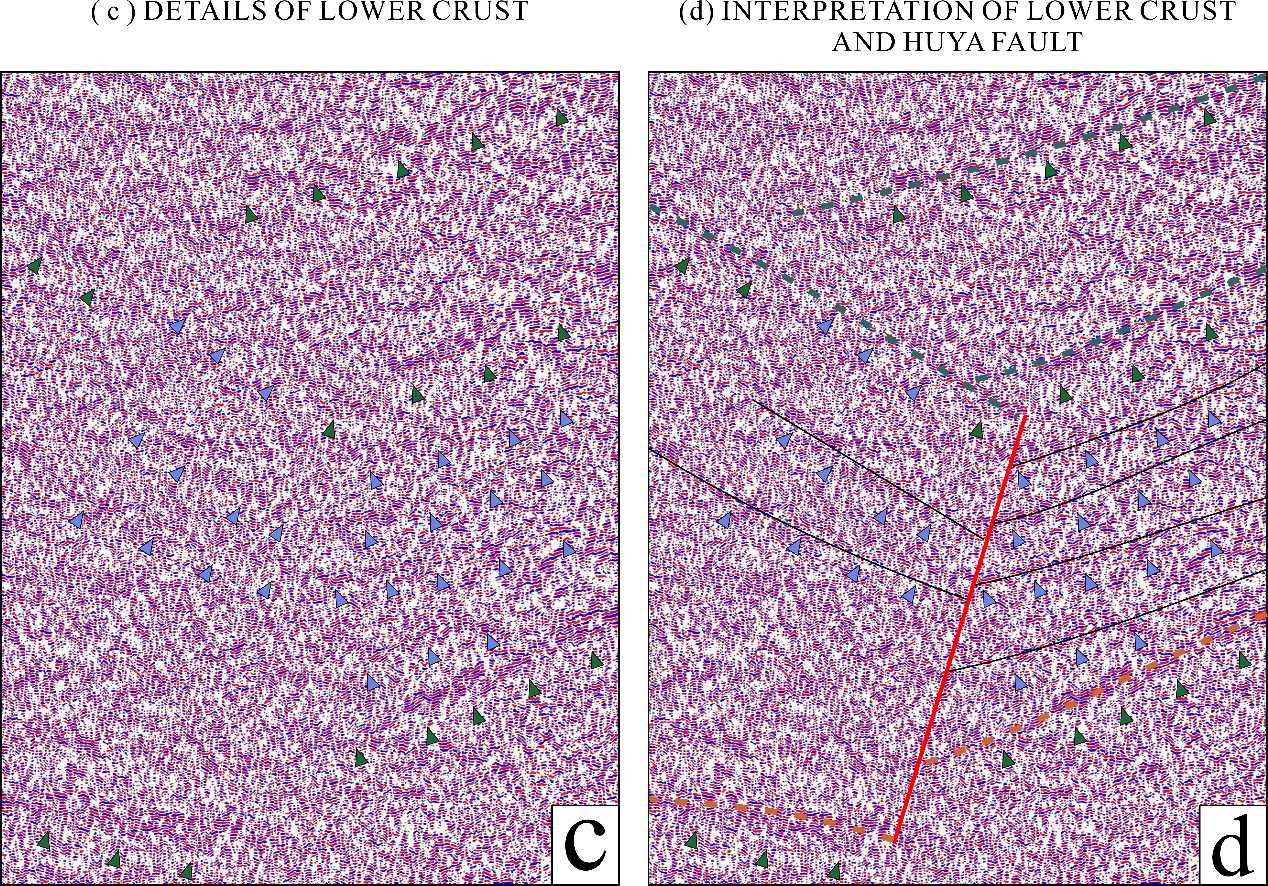
**

**
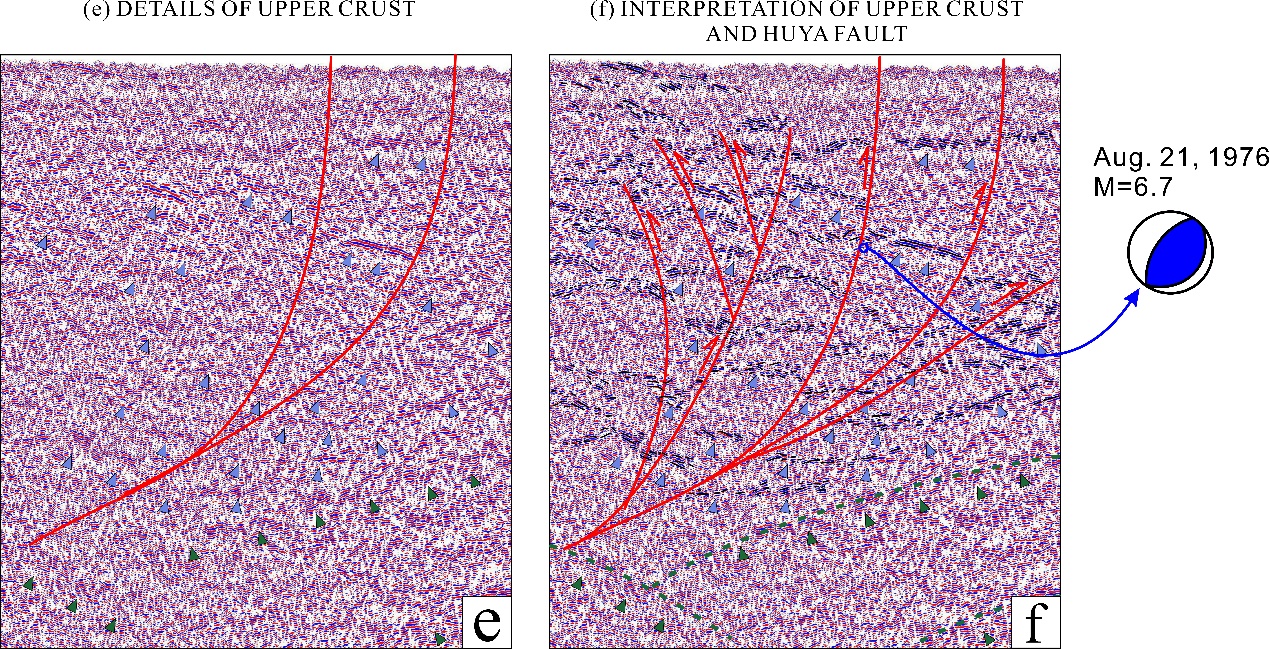
**

**
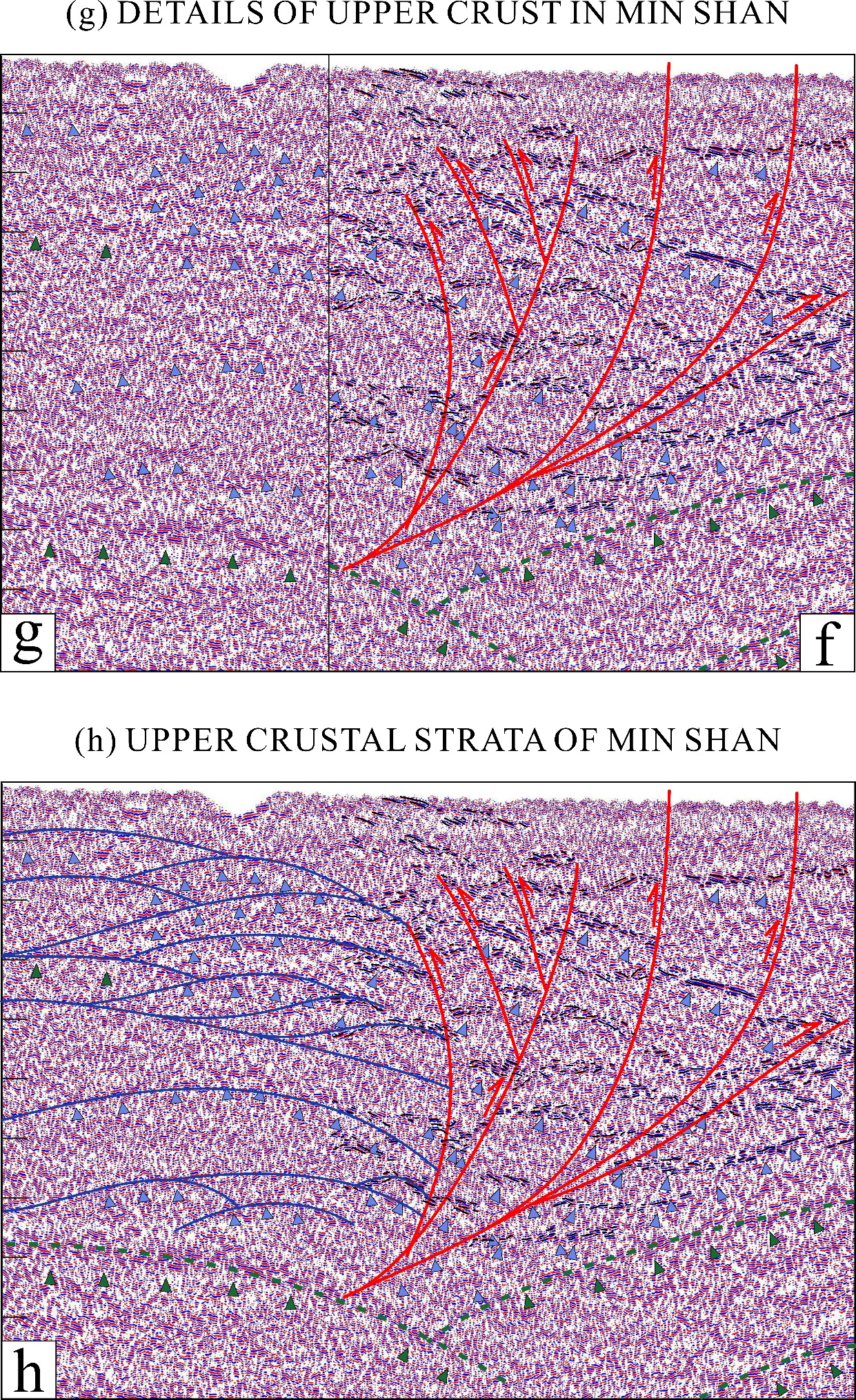
**

**
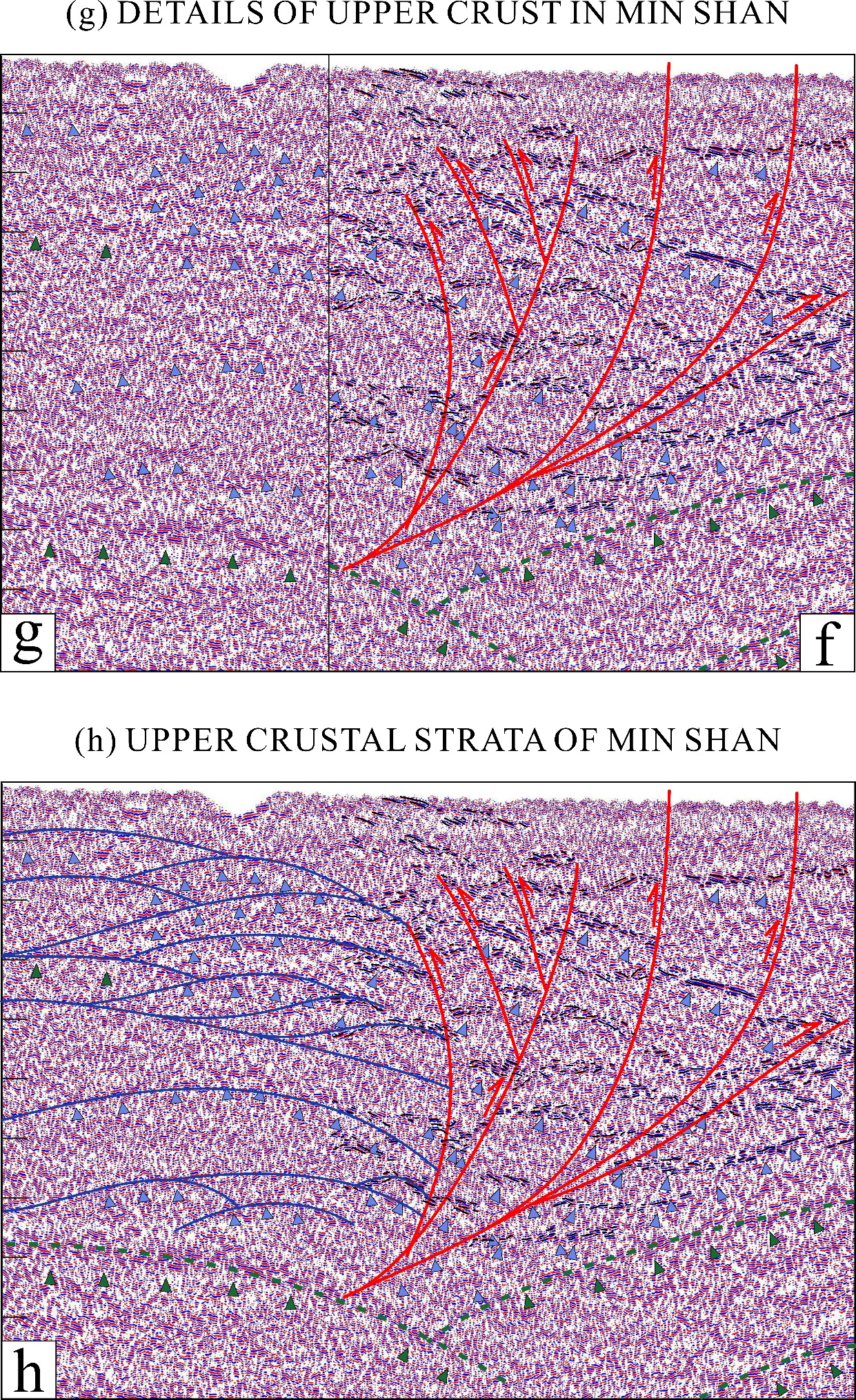
**

**Supplementary Figure S3. | Crustal deformation in the Min Shan region.**

The crustal-scale shortening along the eastern plateau margin is a primary mechanism driving uplift. Decoupled shortening has occurred between the upper and lower crust. This figure shows the uplifting mechanism of the Min Shan region.

**
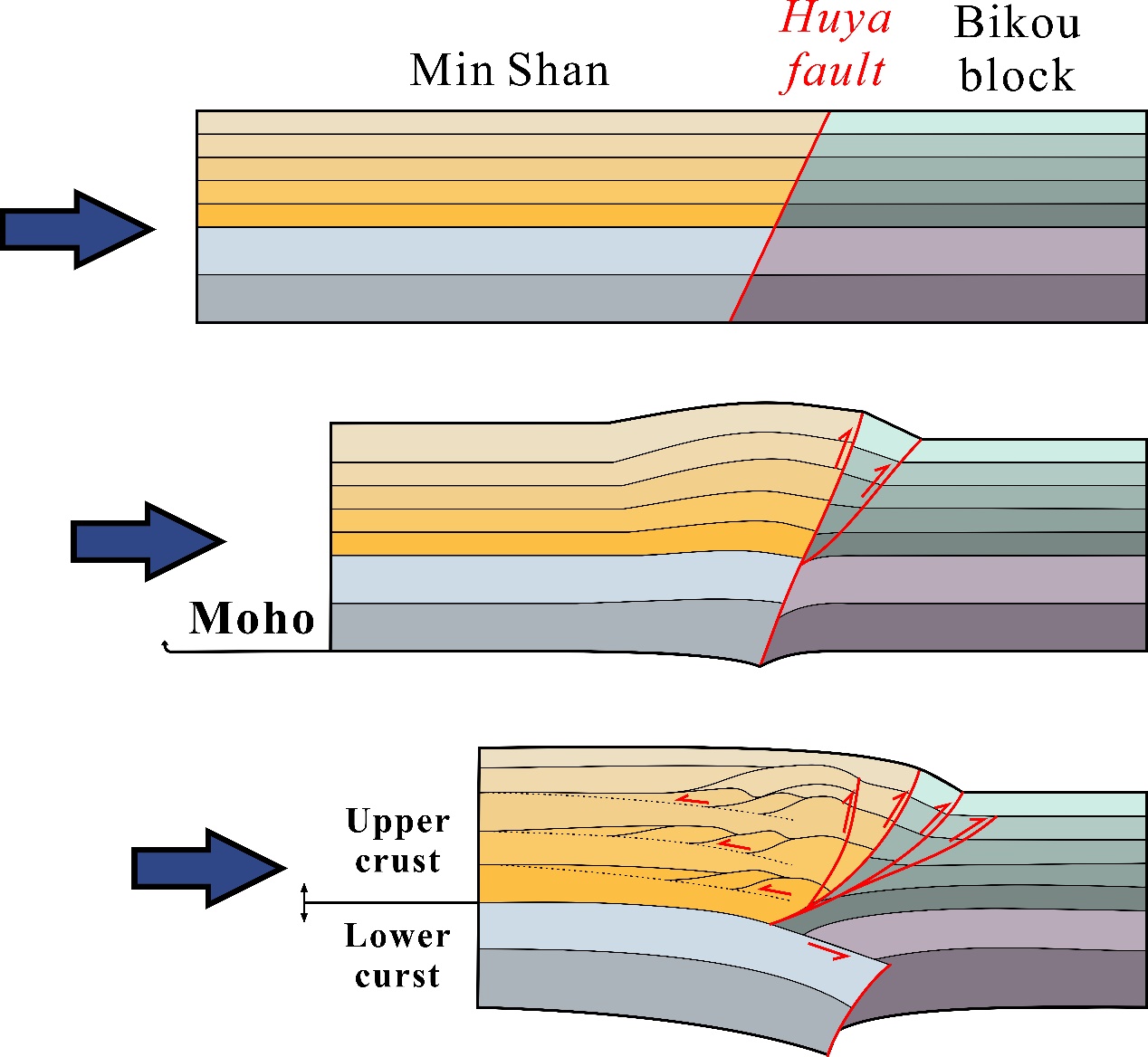
**
